# Perceived social support and quality of life among adolescents in residential youth care: a cross-sectional study

**DOI:** 10.1186/s12955-021-01676-1

**Published:** 2021-01-22

**Authors:** Marianne Tevik Singstad, Jan Lance Wallander, Hanne Klæboe Greger, Stian Lydersen, Nanna Sønnichsen Kayed

**Affiliations:** 1 Regional Centre for Child and Youth Mental Health and Child Welfare (RKBU), Department of Mental Health, Faculty of Medicine and Health Sciences, Norwegian University of Science and Technology (NTNU), Pb 8905 MTFS, 7491 Trondheim, Norway; 2 Psychological Sciences and Health Sciences Research Institute, University of California, Merced, USA; 3 Department of Child and Adolescent Psychiatry, St. Olavs Hospital, Pb 6810 Elgeseter, 7433 Trondheim, Norway

**Keywords:** Adolescents, Residential youth care, Health-related quality of life, Perceived social support, Maltreatment, Polyvictimization

## Abstract

**Background:**

Residential youth care (RYC) institutions aim to provide care and stability for vulnerable adolescents with several previous and present challenges, such as disrupted attachments, wide-ranging adverse childhood experiences, mental health problems, and poor quality of life (QoL). To the best of our knowledge, the present study is the first to provide knowledge of the associations between perceived social support and QoL and to explore the potential moderating effect of perceived social support on QoL for adolescents who have experienced maltreatment and polyvictimization.

**Methods:**

All RYC institutions with adolescents between the ages 12–23 in Norway were asked to participate in the study. A total of 86 institutions housing 601 adolescents accepted the invitation, from which 400 adolescents volunteered to participate. The Child and Adolescent Psychiatric Interview was used to gather information on maltreatment histories and degree of victimization; the Kinder Lebensqualität Fragebogen was used to measure QoL through several domains (overall QoL, physical well-being, emotional well-being, and self-esteem); and the Social Support Questionnaire was used to measure perceived social support. Linear regression and independent samples t-test were used to study the associations between perceived social support and QoL as well as the potential moderating effect of perceived social support in the association between maltreatment history and QoL.

**Results:**

Perceived social support was positively associated with QoL for both girls and boys, with domain-specific findings. A higher number of different types of support persons was associated with overall QoL, emotional well-being, and self-esteem for boys, but only with self-esteem for girls. Individual social support from RYC staff and friends was associated with higher QoL for girls. However, perceived social support did not moderate the association between maltreatment history and reduced QoL for either sex.

**Conclusions:**

This study emphasizes the importance of maintaining social support networks for adolescents living in RYC, the crucial contribution of RYC staff in facilitating social support, and the potential value of social skills training for these vulnerable adolescents. Furthermore, a wider range of initiatives beyond social support must be carried out to increase QoL among adolescents with major maltreatment and polyvictimization experiences.

## Background

Adolescents living in residential youth care (RYC) institutions often have a background characterized by adverse childhood experiences (ACE), including abuse, neglect, and household dysfunction, making them more prone to negative emotional, behavioral, and social developmental outcomes [1–3] as well as lower quality of life (QoL) [4, 5]. Consequently, the professional monitoring and establishment of a positive social climate are important in avoiding negative outcomes [6, 7]. Knowledge of the potential protective factors for vulnerable adolescents' development while living in RYC is generally lacking despite its integral role in providing optimal care and in informing policies and practices for providing high-quality RYC institutions. Perceiving social support can be relevant in this regard; however, adolescents in RYC report lower perceived social support [8] compared to adolescents in the general population. Thus, the aim of the current study is to investigate the associations between perceived social support and QoL for these high-risk adolescents and determine the potential moderating effect of perceived social support on QoL for those with maltreatment and polyvictimization experiences.

### Adolescents living in RYC

Adolescents living in RYC are characterized as a vulnerable population, often having experienced neglect and abuse during their childhood [2, 9]. Such a background can potentially lead to poor interpersonal relationships and feelings of instability and distrust, especially when the traumatic event occurs within the family [10, 11]. RYC placements by the Norwegian Child Welfare Services (CWS) are aimed at adolescents who have faced a wide range of challenges or have been raised in troubled backgrounds, making it reasonable to assume that they have experienced neglect to some extent. A Norwegian study among foster children found that 86.3% had experienced serious neglect [12]. Growing up with ACE, several placements, and disrupted attachments have been associated with behavioral, psychological, social, and educational problems among adolescents [13–15]. During adolescence, the extensive biological, social, and psychological developments [16] are also influenced by both individual and environmental factors [17]. Even though the primary purpose of RYC placements is to support positive development with the provision of a safe and caring environment, the strain caused by the immediate change in residency can disrupt previously established healthy attachments and ultimately negatively impact the adolescents’ mental health, perceived stress, and social relationships [18, 19]. Consequently, these psychosocial strains put them at greater risk for poor QoL [4, 20], mental health problems [21, 22], and low levels of perceived social support [8].

### Quality of life

QoL refers to an individual’s subjective perception of well-being in different life domains. For the adolescent population, a broader coverage of this concept is preferred, including measures of QoL related to family, friends, and school [23]. For this reason, we use the health-related definition of QoL, which views it as “a psychological construct which describes the physical, mental, social, psychological and functional aspects of well-being and function from the patient perspective” [24].

Most of the related research have found that girls report lower QoL compared to boys [4, 25], with one exception for disadvantaged youths, where no sex difference has been found [26]. Past research generally reported decreasing QoL and subjective well-being at younger ages [4, 25]. Moreover, both personal and environmental psychosocial risk factors may influence an individual's sense of well-being, thereby affecting QoL [25]. Previous experiences of maltreatment, mental health problems, and other stressful life events have also been associated with poor QoL [20, 27, 28]. The sparse research on adolescents living in RYC report significantly poorer QoL than adolescents living with their biological families [4, 25]. Jozefiak and Kayed [5] studied the same population as in the current study and found that, compared to the general population, adolescents in RYC reported lower scores in the life-domains of physical well-being (PWB), emotional well-being (EWB), self-esteem and friends, which raise major concerns. Greger and colleagues [20] also found a dose–response relationship between the number of types of ACE and QoL, which has also been reported in other populations [29, 30]. Despite these findings and the fact that several researchers have stated a need for more in-depth investigations of the potential predictors of high-risk adolescents’ QoL [4, 25, 31], research on the potentially moderating factors for QoL among adolescents with experiences of maltreatment and polyvictimization is still lacking.

### Perceived social support

Perceived social support is defined as the availability of people who make one feel cared about, valued, and loved [32]. Having social relationships with others is a basic human need and is important for a healthy development, as early relational experiences affect and form the quality of and expectations in later social relationships [33, 34]. For adolescents in RYC, a previous lack of stable social relationships and reliable care could cause a mistrust of others and insecurity in their present social relationships [3, 11]. However, new social relationships can still develop positively, as previous experiences are not automatically transferred into new social relationships, and the strength of each social relation is person-specific [31, 35]. For adolescents in RYC, identifying the potential possible social support providers is particularly important, as they may require substitute support persons in the case of inadequate parental support.

One study on the same population as the current study found that adolescents in RYC perceive less social support than adolescents in the general population, with mothers, friends, and RYC staff serving as the important social support providers [8]. Additionally, boys in RYC tend to perceive lower social support than girls [36], whereas girls tend to be more available for emotional closeness in social relationships than boys [37, 38]. Social support, however, is especially important for these vulnerable adolescents, as it has been found to reduce feelings of stress and can facilitate successful adaptation to new situations [39, 40]. Social support is also positively associated with well-being [41], adjustment [36], mental health [42, 43], and educational achievement [44]. However, despite the importance of social support and the risks associated with inadequate support, studies on the associations between social support and QoL for adolescents living in RYC remain sparse.

### Quality of life and perceived social support

Social relationships [33, 45] have been found to influence adolescents’ QoL [46], with research suggesting that having a high number of available social resources helps ensure that vulnerable adolescents maintain good QoL. Mendonça and Simões [26] found positive associations between QoL and the availability of social support from multiple sources among socioeconomically disadvantaged youth, but only allowed for three social support categories with poor differentiation among important sources. Alriksson-Schmidt and colleagues [47] found that the availability of several social resources could lead to better QoL for adolescents with mobility disability. However, neither of these studies included adolescents in the out-of-home care setting, nor did they investigate the number of different support persons or individual social support providers.

For adolescents in the general population, family members play a salient role in QoL and overall life-satisfaction [48, 49], especially parents who help in monitoring and developing their communication skills [50]. As adolescents in RYC are separated from their biological families, identifying other adults who can serve as a partial substitute for the lack of parental presence and support, such as the RYC staff [51], is important. The RYC staff can serve as valuable contributors to the overall well-being of adolescents living in RYC [37]. In fact, adolescents who stayed longer in RYC reported higher QoL than those with shorter stays [25], possibly suggesting that secure attachments with the RYC staff can develop over time. Another study found that interpersonal relationships with parents, staff, and friends are the most frequently reported determinants of better overall QoL for adolescents in RYC [52]. However, given the lack of empirical evidence, these hypotheses need further investigation. To the best of our knowledge, no study has investigated the unique effects of parental, friend, or staff support on the QoL of adolescents living in RYC.

While the number of childhood adversities has been found to be positively associated with poorer QoL [20], the potential moderating factors should also be investigated, including perceived social support. In a recent study on the QoL of adolescents in the general population, the association between maltreatment and QoL remained significant, and perceived social support moderated the negative effects of the maltreatment [29]. However, other studies claim that perceiving social support is insufficient as a protective factor for adolescents who have experienced severe child maltreatment and abuse [53, 54]. Currently, the potential moderating effect of perceived social support for high-risk adolescents living in RYC has yet to be adequately investigated.

### Aims of the current study

The current study aims to investigate the associations between perceived social support and QoL, as well as the potential moderating effect of perceived social support on maltreated adolescents’ QoL. Hence, we propose the following hypotheses:

1. Perceived social support from a high number of support persons is associated with better QoL.
2. The association between perceived social support and QoL depends on the individuals from whom the adolescents perceive social support.
3. Perceived social support moderates the negative effects of maltreatment on adolescents’ QoL.

As previous research has established the importance of sex and age in relation to measuring QoL [55], sex and age differences will be controlled for in the current study.

## Methods

### Data

#### Setting

The Norwegian Directorate for Children, Youth, and Family has the responsibility for overseeing the operation of RYC facilities in Norway, where children and adolescents aged 12–23 years (> 18 only if volunteering for placement) are placed according to the Child Welfare Act. These placements are often due to family problems, parents’ inability to provide care, parents’ substance use, or adolescent behavior problems [6, 56]. The adolescents in the current sample reported the following main reasons for their first out-of-home placement: problems between the adolescent and the parents (43.4%), such as constant arguing, disagreements, or violence, and individual adolescent (30.6%) or parental (25.6%) characteristics, referring to extensive problems with, for example, anger or violence, apart from wide ranging mental health problems or issues related to substance use.

Norwegian RYC institutions usually house 3–5 residents at a time, with the aim of providing a home-like, caring environment for the adolescents. As they are not primary treatment facilities, direct services are provided by other community agencies. The institutional staff are responsible for the everyday care of the adolescents and serve as substitute parents as they are the adolescents' primary caregivers while living in RYC. Aside from providing care, monitoring, and support, the staff also initiate participation in school (almost 70% attend school) and leisure activities for the adolescents. For each adolescent, one of the staff members functions as a primary contact with the overall responsibility for the adolescent while living in RYC [57]. The staff either work three shifts per day (daytime, evening, or night shift) or they stay at the institutions for 3–7 days before having a longer period off. The educational backgrounds of the staff members differ, as only 50% are required to have relevant education [57]. Over 90% of the adolescents also have contact with their parents or previous caregivers while living in RYC.

#### Study population

**Fig. 1:**
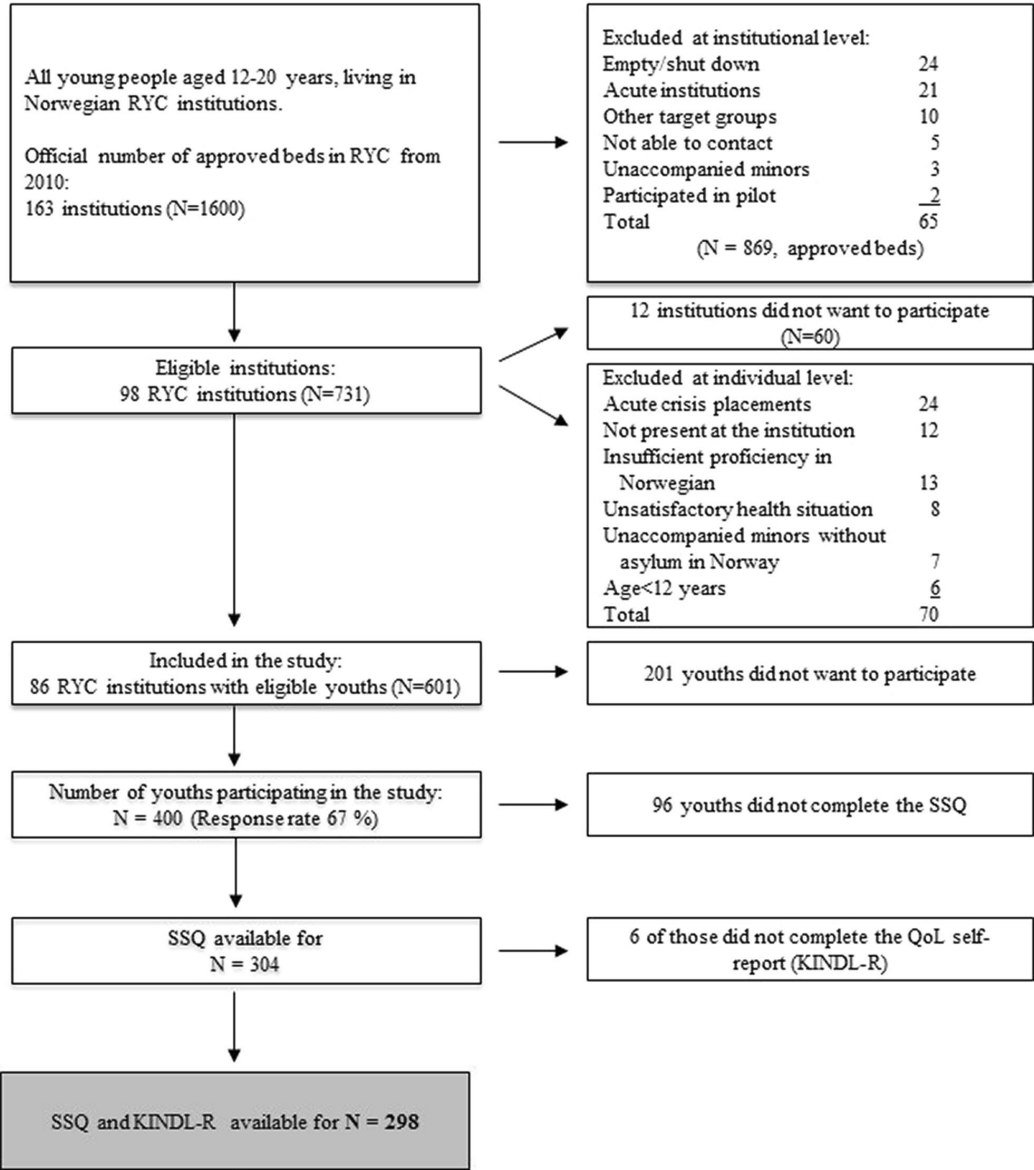
Flow chart for the inclusion of participants. Not able to contact = if the institutional staff did not respond to repeated approaches about participation over a period of several months. There were no significant differences between participating and non-participating institutions with regard to geography and ownership. RYC = Residential Youth Care; SSQ = Social Support Questionnaire; QoL = Quality of Life

The data used in the current study were obtained from the Norwegian research project entitled *Mental Health in Adolescents Living in Residential Youth Care* [21]. All adolescents aged 12–23, living in RYC facilities in Norway, and fulfilling the inclusion criteria, were asked to participate in the study. Exclusion was due to both individual and institutional characteristics, described in detail in Fig. 1. In short, 86 institutions accepted participation (N = 601), whereas 201 adolescents did not give their consent. Anonymous CBCL-scores (Child Behavior Checklist) were collected for the non-participants, making it possible to perform an attrition analysis, which shows the statistically significant representativeness of participants on mental health scores (please see Jozefiak et al. [21] for further information). A total of 400 adolescents agreed to participate in the study, giving a response rate of 67%. Table 1 presents the main characteristics of the sample. Of those included in the study, 304 completed the Social Support Questionnaire (SSQ), 300 completed the Kinder Lebensqualität Fragebogen (KINDL-R), and 298 adolescents completed both questionnaires. Attrition analysis showed that completers and non-completers had similar distributions for sex, age, age at first out-of-home placement, and total CBCL score [see Additional file 1].

**Table 1:** Sample characteristics

	Total sample	Girls	Boys
	N = 400	N = 231 57.8%	N = 169 42.3%
**Age**			
Mean (SD)	16.5 (1.36)	16.7 (1.25)	16.2 (1.47)
12–13	3.5%	0.9%	7.1%
14–16	48.5%	44.1%	54.4%
17–20	48%	55%	38.5%
**Age at first placement**			
Mean (SD)	12.52 (3.88)	12.63 (3.74)	12.37 (4.07)
0–2 years	4.6%	3.5%	6.0%
3–5 years	3.9%	4.0%	3.6%
6–12 years	25%	25.7%	24.1%
13–15 years	49.5%	50.0%	48.8%
16–17 years	17%	16.8%	17.5%
**Number of placements**			
Mean (SD)	3.34 (2.44)	3.61 (2.70)	2.97 (1.98)
1	19.0%	18.1%	20.1%
2	26.3%	22.4%	31.8%
3–5	41.2%	41.4%	41.0%
> 5	13.5%	18.1%	7.1%
**Reason for first placement**			
Problems parent–child	43.4%	45.7%	40.2%
Parental characteristics	25.5%	33.9%	14.2%
Adolescent characteristics	30.6%	27.4%	34.9%
**Attending school/work**	78.5%	72.7%	86.4%
Attending school	68%	62.3%	75.7%
Work practice	7.5%	8.7%	5.9%
Attending work	3.8%	3.0%	4.7%

#### Procedures

All RYC institutions in Norway were randomly arranged in a database, and representative staff were contacted personally by research assistants. In the period between 2011 and 2014, four trained research assistants with comprehensive education and work experience with children and their families carried out the data collection. Adolescents, primary contacts, and leaders at the institutions completed different questionnaires. When necessary, breaks were adapted for the adolescents, and data collection was conducted over two days to minimize the strain. Each adolescent was compensated with 500 NOK, and four randomly chosen adolescents won an I-phone.

### Instruments

#### The Kinder Lebensqualität Fragebogen (KINDL-R)

To measure QoL, we used the Norwegian translation of The Kinder Lebensqualität Fragebogen revised version (KINDL-R) [23], a well-established instrument used in numerous clinical and epidemiological studies. KINDL-R consists of 24 items divided into six subscales: Physical well-being (PWB), Emotional well-being (EWB), Self-esteem, Family, Friends, and School. Each item addresses the child’s experiences over the past week rated on a 5-point scale, ranging from 1 (never) to 5 (always). A sum score is calculated for each subscale and for the overall score, where a higher score indicates better QoL (max = 100). The questionnaire has shown good scale fit and satisfactory internal consistency [58] and test–retest reliability [59]. For the present study, the subscales Family, School, and Friends were excluded. The Family subscale, which include questions related to family life in the past week, was not relevant for the current population. The School subscale was removed for the main analysis, because 29% of the participants were not attending school, although additional analyses were conducted separately for the participants who were currently enrolled (N = 193). The Friend subscale was removed due to conceptual overlap with the SSQ (e.g., “I was a success with my friends” and “I got along well with my friends”).

#### Social Support Questionnaire (SSQ)

The Social Support Questionnaire measures three aspects of perceived social support, including perceived number of different types of support persons (SSQ-N), social support satisfaction (SSQ-S), and perceived social support from different social support providers. As the satisfaction scale only measures satisfaction with the perceived support in each situation, not individually for each provider, and that the adolescents are generally satisfied with the support perceived [8], we chose not to include this scale in our analyses. Instead, in the current study, we used a short 5-item version [60] developed from the original 27-item version [32]. Briefly, the questionnaire examines who the adolescents can turn to (nine possible support persons) in five hypothetical situations, including different social support domains. First, the SSQ-N score is calculated by counting the number of different types of support persons listed over the five items. This score is then divided by the number of items to exclude overlapping counts of the support persons for the overall SSQ-N score. This score measures the perceived breadth of the respondents' social support network. Second, perceived social support from different providers can be investigated separately and compared to those adolescents not perceiving support from the same group of providers. More detailed information on the SSQ is given in Singstad et al. [43]. The internal consistencies for the scores in the currently used version of the SSQ were α = 0.79 for SSQ-N and α = 0.76 for SSQ-S.

#### Childhood adversity

Information about childhood adversity was drawn mainly from selected questions from a semi-structured psychiatric interview (The Child and Adolescent Psychiatric Assessment/CAPA). In addition, a measure of household dysfunction was created based on information from a questionnaire completed by the adolescents. Those who confirmed that their parents had a history of mental health problems, often got drunk or used drugs, or that they had been removed from the family home because of parental crime, alcohol or drug abuse, or psychiatric problems received a positive score on household dysfunction. We constructed a scale wherein the numbers of types of adversities were added. These adversities included the following: *witness of violence, victim of physical violence, victim of family violence, victim of sexual abuse, and household dysfunction*. Greger et al. [2] provided specific information about childhood adversity in the current sample.

### Statistical analyses

We used linear regression analyses with the overall QoL score and each of the three subscale scores, separately, as dependent variables, with the overall SSQ-N score serving as the covariate, adjusting for age. Independent samples t-test was used to investigate mean level differences in overall QoL and for each subscale score dependent of indications of support from each type of social support provider. To investigate the possible moderating effect of a perceived social support to maltreated adolescents’ QoL, we used linear regression with overall QoL as the dependent variable as well as the social support variable and the childhood adversity scale and their interactions as covariates, adjusting for age. The normality of residuals was checked by visual inspection of the Q-Q plots [61]. All analyses were conducted separately for girls and boys.

These analyses were performed using SPSS version 26. Results are regarded statistically significant where *p* values < 0.05. We report 95% confidence intervals (CI) where relevant.

### Ethics

The project was approved by The Norwegian Regional Committee for Medical and Health Research Ethics (Project 2014/1516). The approved procedures were used in the recruitment of participants, and all participants (including the primary caregiver for those under the age of 16) had to sign an informed consent form before participation.

## Results

### Quality of Life and Breath of Support Network

As detailed in Table 2, for girls, a higher number of different types of support persons (overall SSQ-N) was significantly associated only with higher self-esteem QoL (*p* = 0.014). For boys, significant associations were found with higher overall QoL (*p* = 0.005), EWB (*p* = 0.020), and self-esteem (*p* = 0.001). A separate analysis on those participating in school (N = 193) revealed no association with the school QoL.

**Table 2:** Associations between QoL domains and overall SSQ-N score

QoL-score	SSQ-N					
	Girls			Boys		
	b	95% CI	*p*	b	95% CI	*p*
Overall QoL	2.20	[− .28 to 4.68]	.08	3.31	[1.05 to 5.58]	**.005**
PWB	1.71	[− 1.22 to 4.65]	.25	1.37	[− 1.11 to 3.85]	.28
EWB	1.27	[− 1.58 to 4.12]	.38	3.11	[.50 to 5.72]	**.020**
Self-esteem	3.62	[.75 to 6.50]	**.014**	5.46	[2.15 to 8.78]	**.001**

All analyses are adjusted for age

QoL, Quality of Life; overall SSQ-N score, total number of different types of support persons; PWB, Physical Well-Being; EWB, Emotional Well-Being

Bold: *p* < 0.05

### Quality of Life and Different Providers of Social Support

As detailed in Table 3, perceiving social support from parents was not significantly associated with higher overall QoL nor for any subscale for either girls or boys. Girls perceiving staff support reported significantly higher self-esteem compared to those who did not perceive staff support (*p* = 0.038). For boys, perceiving social support from staff was not significantly associated with any of the QoL scores. Whereas perceiving friend support was significantly associated with an increase in all QoL scores for girls, including overall QoL (*p* = 0.002), PWB (*p* = 0.012), EWB (*p* = 0.010), and self-esteem QoL (*p* = 0.003), no increase in the QoL scores for boys were found.

**Table 3:** QoL scores depending on perceived social support from different providers, separately for girls and boys

Support provider and QoL-score	Girls						Boys					
	Perceived support						Perceived support					
	No		Yes		Difference		No		Yes		Difference	
	n	Mean	n	Mean	Estimate [95% CI]	*p*	n	Mean	n	Mean	Estimate [95% CI]	*p*
**Mother**												
Overall QoL	55	49.36	114	50.26	− .900 [− 7.85 to 6.05]	.80	39	64.26	90	67.11	− 2.844 [− 10.00 to 4.31]	.43
PWB	55	50.68	114	49.12	1.559 [− 6.53 to 9.65]	.70	39	67.15	90	70.56	− 3.408 [− 11.04 to 4.22]	.38
EWB	55	57.84	114	59.70	− 1.863 [− 9.80 to 6.07]	.64	39	69.55	90	72.15	− 2.602 [− 10.82 to 5.61]	.53
Self-esteem	55	39.55	114	41.94	− 2.395 [− 10.49 to 5.70]	.56	39	56.09	90	58.61	− 2.521 [− 13.10 to 8.06]	.64
School^a^	38	48.87	65	50.66	− 1.782 [− 9.41 to 5.85]	.64	30	61.69	60	67.90	− 6.215 [− 14.09 to 1.66]	.12
**Father**												
Overall QoL	97	48.71	72	51.65	− 2.938 [− 9.51 to 3.63]	.38	60	65.90	69	66.55	− .643 [− 7.25 to 5.96]	.85
PWB	97	48.52	72	51.13	− 2.610 [− 10.27 to 5.05]	.50	60	69.90	69	69.20	.693 [− 6.35 to 7.74]	.85
EWB	97	56.64	72	62.41	− 5.777 [− 13.25 to 1.69]	.13	60	71.25	69	71.47	− .217 [− 7.79 to 7.36]	.96
Self-esteem	97	40.98	72	41.41	− .427 [− 8.10 to 7.25]	.91	60	56.56	69	58.97	− 2.405 [− 12.15 to 7.34]	.63
School^a^	63	48.97	40	51.62	− 2.655 [− 10.20 to 4.89]	.49	43	64.35	47	67.19	− 2.839 [− 10.35 to 4.67]	.46
**Staff**												
Overall QoL	63	47.16	106	51.63	− 4.475 [− 11.18 to 2.23]	.19	41	65.35	88	66.67	− 1.321 [− 8.39 to 5.75]	.71
PWB	63	49.90	106	49.47	.431 [− 7.41 to 8.27]	.91	41	67.53	88	70.45	− 2.924 [− 10.46 to 4.61]	.44
EWB	63	55.56	106	61.20	− 5.647 [− 13.29 to 2.00]	.15	41	71.80	88	71.16	.634 [− 7.48 to 8.75]	.88
Self-esteem	63	36.01	106	44.22	− 8.210 [− 15.96 to − .46]	**.038**	41	56.71	88	58.38	− 1.673 [− 12.12 to 8.77]	.75
School^a^	34	44.27	69	52.82	− 8.555 [− 16.21 to − .90]	**.029**	27	66.83	63	65.40	1.431 [− 6.78 to 9.64]	.73
**Friend**												
Overall QoL	16	34.24	153	51.61	− 17.362 [− 28.17 to − 6.56]	**.002**	15	62.22	114	66.78	− 4.554 [− 14.80 to 5.69]	.38
PWB	16	34.77	153	51.18	− 16.419 [− 29.13 to − 3.71]	**.012**	15	64.58	114	70.18	− 5.592 [− 16.52 to 5.33]	.31
EWB	16	44.14	153	60.66	− 16.521 [− 28.97 to − 4.07]	**.010**	15	69.58	114	71.60	− 2.018 [− 13.80 to 9.77]	.74
Self-esteem	16	23.83	153	42.97	− 19.146 [− 31.77 to − 6.52]	**.003**	15	52.50	114	58.55	− 6.053 [− 21.19 to 9.09]	.43
School^a^	7	26.79	96	51.69	− 24.905 [− 38.70 to − 11.11]	**.001**	12	59.95	78	66.74	− 6.787 [− 17.77 to 4.19]	.22

Respondents are included in the groups «No support» and «support» regarding on whether they perceive the actual support person as a source of support or not. The section for school participants only include overall QoL (consisting of the subscales PWB, EWB, Self-esteem, and School)

QoL, Quality of Life; CI, confidence interval; PWB, Physical Well-Being; EWB, Emotional Well-Being

^a^Reported only by those enrolled in school, and for overall QoL only

Bold: *p* < 0.05

Additional analyses for the school participants’ reports on the School subscale for girls found associations between overall QoL and perceiving staff support (*p* = 0.029) and friend support (*p* = 0.001). No significant associations were found for perceived social support from individual support providers and overall QoL for boys in the school-participant group.

### Moderating effect of perceived social support on maltreated adolescents’ QoL

Table 4 presents the results from analyses to test the moderation by different social support aspects in the relationship between childhood adversity and overall QoL. As none of the relevant interaction terms were statistically significant, and the corresponding confidence intervals were wide, the results did not confirm moderation by either overall SSQ-N or perceiving support from any of the sources considered here.

**Table 4:** Potential moderation by social support in the relationship between childhood adversity and overall QoL

	Overall QoL							
	Girls (N = 148)				Boys (N = 112)			
	b	95% CI		*p*	b	95% CI		*p*
		Lower	Upper			Lower	Upper	
CAS	− 4.37	− 7.34	− 1.40	**.004**	− 2.76	− 6.13	.618	.11
Overall SSQ-N*CAS	− 1.28	− 3.47	.92	.25	− .97	− 3.34	1.41	.42
Mother supp.*CAS	− 3.12	− 9.41	3.17	.33	− 2.56	− 9.68	4.57	.48
Father supp. * CAS	− 2.63	− 8.63	3.37	.39	− 4.81	− 11.65	2.03	.17
Staff supp. * CAS	− 2.07	− 8.66	4.53	.54	.09	− 6.88	7.06	.98
Friend supp. * CAS	3.81	− 4.64	12.27	.37	− 3.65	− 15.57	8.28	.55

The first line shows the regression coefficient for the CAS as independent variable. The rest of the table shows the coefficient for the interaction between a social support variable and the CAS, in an analysis including these variables and their interaction. All analyses are adjusted for age

QoL, Quality of Life; overall SSQ-N, total number of different types of support persons; CAS, Childhood Adversity Scale

Bold: *p* < 0.05

## Discussion

Our results showed that QoL is associated with perceived social support for adolescents living in RYC, although there are differences between girls and boys. For the number of different types of support persons, most associations to QoL were found for boys, namely, for overall QoL, EWB, and self-esteem. For girls, significant associations were only observed for self-esteem. For different providers of support, significant associations were found for girls between the self-esteem and perceiving staff support and for all QoL aspects when perceiving friend support. For boys, no significant associations were found in relation to different providers of support. In addition, perceiving social support did not moderate the negative effects of previous experiences of maltreatment and polyvictimization on adolescents’ QoL.

### Quality of life and breath of support network

For boys living in RYC, a higher number of different types of support persons is associated with better QoL in several domains, including overall QoL, EWB, and self-esteem. This is not a surprising finding, as EWB covers the degree of happiness, loneliness, and insecurity. Boys have previously been reported to seek activity in their interactions and seem to benefit the most from receiving social support through group activities [62]. Therefore, having several different support providers who are available in multiple areas can, for example, improve their degree of happiness and contribute to less feelings of loneliness. This also applies to the association with self-esteem, which measures, for example, feelings of worth and satisfaction with one`s own performance. The presence of positive relationships and having a sense of acceptance and being valued through supportive relationships are likely to increase the adolescents’ self-esteem [32, 63]. This also applies to girls based on the significant association found between self-esteem and the breadth of their social network.

### Quality of life and different providers of social support

For adolescent girls' QoL, perceiving social support from some specific social support providers (i.e., institutional staff and friends) appears important. Girls perceiving staff support reported higher self-esteem compared to those without this support, although the result is not highly significant. One can assume that institutional staff have an important contribution in supporting these girls in everyday life, possibly fostering a belief in themselves and their own capacity. As girls report a higher need of closeness and one-to-one interactions in their supportive relationships compared to boys [37, 64], the presence and stability of the institutional staff are crucial in this context. Whereas parents most often are the important contributors to children’s self-esteem [65], it might be that the institutional staff can substitute for the lack of parental presence for girls while they live in RYC, which would be encouraging.

Additional analyses on the school participants found significant associations between perceived staff support and overall QoL. Adolescents attending school while in RYC are younger (mean age = 16.0) than those who are not attending school (mean age = 16.9, *p* < 0.01). Therefore, given that younger adolescents are often in need of significant support from their primary caregivers [8, 66], it is not surprising that the RYC staff are important contributors to these girls’ feelings of security and being cared for in the absence of parental support [8, 67]. The fact that the RYC staff can promote positive outcomes, such as higher well-being for adolescents living in RYC, is consistent with previous research [40, 68, 69]. It is a well-known fact that friends become increasingly important with higher age [66, 70], so the significant associations between friend support and QoL across all domains for girls are not surprising, as they coincide with previous research [52]. Girls mostly report valuing closeness and the emotional aspects of social support through one-to-one interactions [38, 62, 70], so that they consider being cared for, valued, and accepted by friends as particularly important during adolescence [8, 70]. This is also associated with better QoL for girls. For boys, perceiving social support from individual providers did not appear to play a role in their QoL.

### The potential moderating effect of perceived social support on maltreated adolescents QoL

We did not find any evidence to support the hypothesis that perceived social support moderated the effect of maltreatment on these adolescents’ QoL. Adolescents living in RYC are particularly vulnerable, as they have simultaneous experiences of maltreatment, household dysfunction, and out-of-home placements. We know that they report poor QoL compared to peers in the general population and that there is a dose–response relationship between the number of events and poorer QoL [20]. Previous research have found that a higher number of childhood adversities reduces the likelihood of social support being a moderator for the adolescents’ poor QoL [53, 54]. The current lack of statistically significant results concerning both the number of different types of support persons and individual support providers strengthens the knowledge of the critical long-term consequences of growing up with child maltreatment and household dysfunction [1, 20]. Perceiving social support does not seem by itself to protect these vulnerable adolescents’ QoL while living in RYC.

### Limitations

The cross-sectional nature of this study limits the interpretation of our results. First, the current study cannot state the causal relationships between perceived social support and QoL; it can only indicate the need for a longitudinal study of these associations. Second, more background variables concerning the respondents, such as mental health before and at the time of placement, age at each placement, length of stay in each out-of-home placement, and frequency of contact with significant others outside the RYC, would have been beneficial and could have provided deeper insights. In addition, we did not have the opportunity to include parents as respondents in measuring QoL, because the adolescents did not live at home, which led to the exclusion of considering family functioning. School functioning was also excluded, because it did not apply to a portion of those in RYC. Furthermore, we lacked measurement of QoL for about 25% of the adolescents participating in the overall study, but the analytic sample appeared to be representative because distributions of sex, age, and internalizing and externalizing mental health problems did not differ between the completers and the non-completers.

The SSQ also has some limitations. In measuring perceived social support, additional sources of social support could be addressed, including the opportunity to add unnamed sources. This would have provided deeper insights into the role of different social support providers in improving the QoL of adolescents in RYC.

### Future practice

As research on the associations between perceived social support and QoL for this vulnerable group of adolescents is generally lacking, results should be helpful in developing practices to provide the best care possible in RYC and in planning further research. Given that maltreatment is common among these adolescents [20], it is reasonable to assume a high prevalence of social skill deficit in this group [71]. As social support is associated with increased QoL, more specifically to a wider social network for boys and for friend and staff support for girls, further development of social skills and the conduct of social skills training should be prioritized in RYC. An increase in these adolescents’ social skills might contribute to both maintaining and establishing social relationships while living in RYC. The RYC staff also have an important role in ensuring the maintenance of the already established social networks for these adolescents, so they can benefit from their positive effects while living in RYC. At the same time, one should be cautious regarding the possible negative influence some friends could have on the adolescents’ behaviors [72]. Arenas for socialization, preferably close to the institutions, should be prioritized. Finally, the length of the residential stays influence adolescents’ well-being, adjustment, and relations to the staff [36]. Thus, disruptions in the RYC placement should be prevented.

Given that perceiving social support does not appear by itself to moderate the negative effects of maltreatment and polyvictimization on these adolescents’ QoL, other initiatives should be explored to help them improve their QoL. Knowledge about other factors that could moderate the association between the negative effects of maltreatment and QoL would be highly valuable, as it can broaden the scope of possible solutions to help these vulnerable adolescents. Finally, these findings highlight the need for more research on potentially protective factors for adolescents in RYC.

## Conclusions

Adolescents living in RYC typically have several previous negative life experiences and face a high prevalence of current difficulties and challenges, which are likely to have a negative effect on their QoL. Therefore, increasing these adolescents’ QoL should be a priority for national authorities as they work on providing the best care possible in RYC. The current study suggests that adolescents’ social support network has an important contribution to their QoL. However, various aspects of social support appear differentially beneficial for girls and boys. A larger network of different types of support persons appears significant for boys, whereas specific providers of social support (especially friends) providing one-to-one interactions appear most beneficial for girls. In summary, these findings expand our current knowledge of the potential critical factors contributing to adolescents’ QoL while living in RYC facilities.

## Supplementary Information

**Additional file 1.** Descriptive statistics for completers and non-completers of the Social Support Questionnaire.

## Data Availability

The datasets used and analyzed during the current study are available from the corresponding author on reasonable request.

## Abbreviations

ACE: Adverse childhood experiences
CAPA: The Child and Adolescent Psychiatric Assessment
CBCL: Child Behavior Checklist
CWS: Child welfare services
EWB: Emotional well-being
KINDL-R: The Kinder Lebensqualitat Fragebogen revised version
PWB: Physical well-being
QoL: Quality of life
RYC: Residential youth care
SSQ: Social support questionnaire
Overall SSQ-N: Total number of different types of support persons
SSQ-S: Social support satisfaction
