# Supplementary material for: Perceived social support and quality of life among adolescents in residential youth care: a cross-sectional study
Source: Health Qual Life Outcomes. 2021 Jan 22;19:29. doi: 10.1186/s12955-021-01676-1 (PMC7821657; doi:10.1186/s12955-021-01676-1)
Supplement: Supplementary file 1 — Additional file 1. Descriptive statistics for completers and non-completers of the Social Support Questionnaire. [file 12955_2021_1676_MOESM1_ESM.docx]

Additional file 1

Attrition analysis: Descriptive statistics for completers and

non-completers of the Social Support Questionnaire.

|  | **n(%) or mean(SD)** | | |
| --- | --- | --- | --- |
|  | **Completers**  **(n=304)** |  | **Non-completers**  **(n=96)** |
| Female sex | 173(57%) |  | 58(60%) |
| Age | 16.80(1.38) |  | 16.63(1.32) |
| Age at first out-of-home placement | 12.42(4.04) |  | 12.85(3.32) |
| Total CBCL-score | 79.00(20.15) |  | 83.80(21.53) |
